# A case of laparoscopic distal gastrectomy and median arcuate ligament release for a gastric cancer patient with median arcuate ligament syndrome

**DOI:** 10.1186/s40792-022-01517-3

**Published:** 2022-08-18

**Authors:** Sho Ueda, Masato Maeda, Kenta Nishitani, Tada Seiichiro, Keisuke Kawamorita, Tomoyasu Takayanagi, Toshiki Kobayashi, Yosuke Hashimoto, Kei Yonezawa

**Affiliations:** Department of Digestive Surgery, Shizuoka City Shizuoka Hospital, 10-93, Ohtemachi, Aoi-ku, Shizuoka, 420-8630 Japan

**Keywords:** The median arcuate ligament syndrome, Gastric cancer, Laparoscopic surgery

## Abstract

**Background:**

The median arcuate ligament syndrome (MALS) is a disease in which the celiac artery is compressed by the arcuate ligament and causes stenosis. If abdominal pain or an aneurysm is observed in the head of the pancreas, it is necessary to release the arcuate ligament, and recently laparoscopic surgery has been reported. However, the indication for treatment in asymptomatic cases is unknown. The treatment for asymptomatic MALS in patients with gastric cancer who are indicated for surgery is also novel.

**Case presentation:**

A 70-year-old female was found with early gastric cancer in the middle body of the stomach. An enhanced CT scan showed no metastasis, but a gallstone and stenosis of the celiac artery due to the MALS were found. The patient underwent releasing median arcuate ligament after lymph node dissection. A median arcuate ligament was located on the ventral side of the left gastric artery stump, and the celiac artery was exposed when cutting it off. The operation time was 4 h and 59 min, and the bleeding was 6 ml. It took about 5 min to dissect the medial arcuate ligament. The postoperative course was satisfactory, and the patient was discharged 7 days after the operation. CT scan and 3-D CT angiography were performed about 2 months after the operation, and the findings revealed that the celiac artery's stenosis resolved.

**Conclusion:**

The patient underwent laparoscopic gastrectomy and simultaneously the median arcuate ligament release under an excellent visual field. Therefore, median arcuate ligament release may be considered if MALS is found in a gastrectomy case.

## Background

The median arcuate ligament syndrome (MALS) is a disease in which the celiac artery is compressed by the arcuate ligament and causes stenosis. The syndrome was first described by Harjola in 1963 and is typically associated with intense postprandial abdominal pain, weight loss, and nausea and vomiting [1]. Since the first surgical series published by Dunbar in 1965, the treatment of choice has been the release of the median arcuate ligament. In 2000 [2], Roayaie reported the first case of laparoscopic arcuate ligament release, and since then [3], this approach has been written by many surgical teams. If abdominal pain or an aneurysm is observed in the head of the pancreas, it is necessary to release the arcuate ligament. However, the indication for treatment in asymptomatic cases is unknown. The treatment for asymptomatic MALS in patients with gastric cancer who are indicated for surgery is also novel.

## Case presentation

**Fig. 1:**
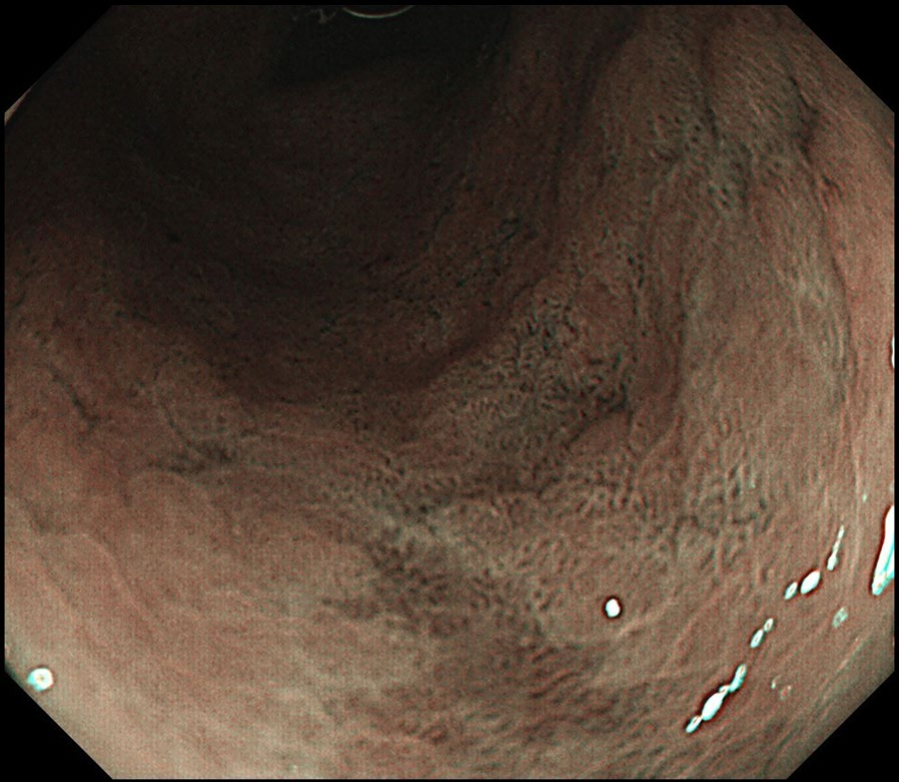
Upper gastrointestinal endoscopy showed an extensive 0–IIc lesion in the middle part of the gastric body

**Fig. 2:**
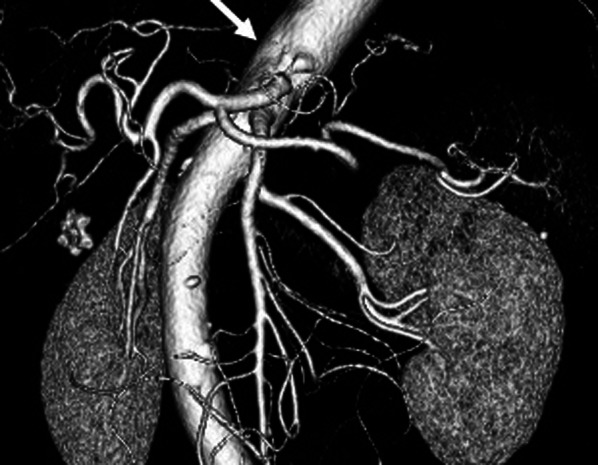
3-D CT angiography showed stenosis of the celiac artery due to MALS (allowed)

A 70-year-old Japanese female was referred to our hospital for early gastric cancer during a medical examination. She underwent a gastroscopy, and the early gastric cancer was found in the middle part of the gastric body (Fig. 1). Although the diagnosis was intramucosal gastric cancer, the lesion was widespread and the histological type was poorly differentiated adenocarcinoma, so surgical resection was indicated. Enhanced CT scan and 3-D CT angiography showed no metastasis, but a gallstone and stenosis of the celiac artery due to the MALS were found (Fig. 2). An aneurysm in the head of the pancreas was not detected. The preoperative stage of gastric cancer was defined as the cT1aN0M0 stage IA, according to the TNM, 8th edition. The patient's height and weight were 160.0 cm and 55.0 kg, respectively. She did not have abdominal pain or symptoms of MALS.

**Fig. 3:**
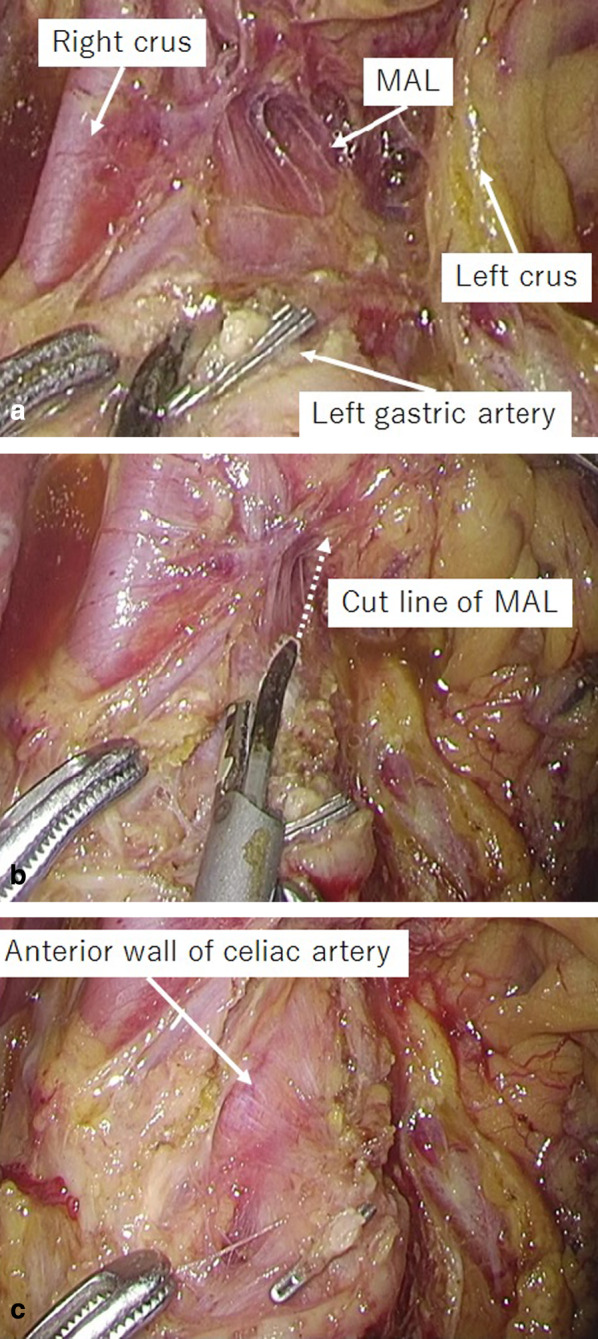
**a** The median arcuate ligament (MAL) was found on the ventral side of the left gastric artery stump. **b** An ultrasonic coagulation cutting device was used to release the MAL. **c** The anterior wall of the celiac artery was exposed

The patient underwent laparoscopic distal gastrectomy with D1 dissection and R-Y reconstruction, cholecystectomy, and median arcuate ligament release. Median arcuate ligament releasing was started after No.9 lymph node dissection. The median arcuate ligament was found on the cranial side of the left gastric artery stump, and the anterior wall of the celiac artery was exposed when cutting it off (Fig. 3). The total operation time was 4 h and 59 min, and the bleeding was 6 ml. It took about 5 min to release the medial arcuate ligament.

The patient's postoperative course was uneventful, and she was discharged home on postoperative day 7.

The pathological stage was diagnosed as pT1aN1M0 stage IA by microscopic examination.

**Fig. 4:**
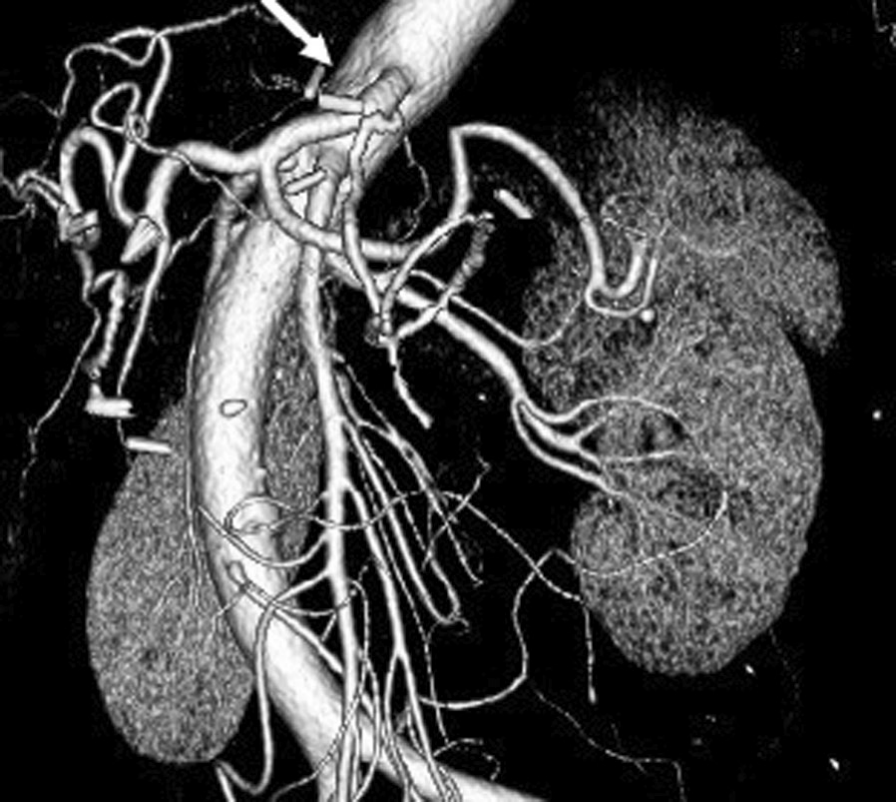
3-D CT angiography was performed about 2 months after the operation, and it was confirmed that the stenosis of the celiac artery due to MALS had been resolved (allowed)

CT scan and 3-D CT angiography were performed about 2 months after the operation, and the findings revealed that the stenosis of the celiac artery due to MALS had been resolved (Fig. 4).

## Discussion

The MALS was first described by Harjola in 1963 [1] and consisted of the extrinsic compression of the celiac trunk by fibers of the median arcuate ligament. MALS causes abdominal symptoms such as postprandial epigastric abdominal pain, weight loss, and abdominal bruit. In 1965, Dunbar reported the release of the median arcuate ligament in 13 patients with promising results [2]. Subsequently, in 2000, Roayaie reported the first case of laparoscopic arcuate ligament release [3]. Recently, laparoscopic management of MALS has been reported. Its advantages include a relatively short operative time, less pain and blood loss, shorter hospital stay, early oral intake initiation, low possibilities of infections, and better visualization of the surgical field [4]. In 2007, Jaik reported the first robot-assisted approach [5], and a similar technique has been reported with excellent results [6, 7].

Symptomatic MALS is an indication of median arch ligament release. Surgical or endovascular treatment indicates asymptomatic MALS with aneurysms in collateral vessels. However, the indication for treatment in asymptomatic cases is unknown. Likewise, the treatment for asymptomatic MALS in patients with gastric cancer who are indicated for surgery is also unknown. As far as we can find in PubMed, there is only one case of gastric cancer complicated with MALS, in which laparoscopic distal gastrectomy and median arch ligament release were performed simultaneously [8]. The patient in this literature was a 62-year-old woman with asymptomatic MALS who underwent median arch ligament release at the same time as her gastric cancer surgery because adhesions would make surgery difficult if surgery for MALS were needed after gastrectomy. In our case, the patient was a 70-year-old woman with asymptomatic MALS who underwent a median arch ligament release performed simultaneously with laparoscopic gastrectomy for the same reason because of the possibility that she would need MALS surgery in the future.

Asymptomatic MALS is often followed up, and we did not find any article reporting the frequency of developing abdominal pain or pancreatic head aneurysms in the future. Therefore, the frequency is probably low, and elderly patients will likely die of other diseases while remaining asymptomatic. Consequently, we believe prophylactic surgery for asymptomatic MALS should be reserved for gastric cancer surgery only in elderly patients with many residual diseases or risk factors, such as liver cirrhosis. Although the patient, in this case, was rather elderly (70 years old), we judged that she had no additional illnesses and few risk factors.

In this case, gastric cancer surgery performed was distal gastrectomy with D1 dissection and R-Y reconstruction; D1 dissection does not require No.9 lymph node dissection, but in this case, No.9 lymph node was dissected when the median arch ligament was exposed, providing an excellent visual field. Most gastric cancers indicated for surgery require D1 + or D2 dissection, and we believe that simultaneous MALS surgery would be more reasonable to dissect the No.9 lymph node. In addition, R-Y or B-II reconstruction may make reoperation for MALS easier than B-1 reconstruction, in which the remnant stomach is attached to the celiac axis. In this case, the patient underwent R-Y reconstruction, and if No.9 lymph node had been preserved, reoperation for MALS might have been possible without solid adhesions.

The patient underwent median arch ligament release simultaneously with laparoscopic gastrectomy under excellent visual field in short time. When MALS is found concurrent with gastric cancer, simultaneous surgery should be considered if symptomatic. Even if MALS is asymptomatic, if the patient is young, the release of the median arch ligament may be necessary for the future. The patient has few risk factors and requires D1 + or D2 dissection, and simultaneous MALS surgery may be considered. If simultaneous MALS surgery is not performed, R-Y or B-II reconstruction may be preferable instead of B-I reconstruction to avoid adhesions. The safety of laparoscopic surgery for both gastrectomy and median arch ligament resection has been reported, and simultaneous laparoscopic surgery is also considered acceptable. Given the widespread use of robotic surgery for gastric cancer and the good results of robotic surgery for median arch ligament release, simultaneous surgery may be considered in robotic surgery.

## Conclusions

Median arch ligament release performed simultaneously with laparoscopic gastrectomy had an excellent visual field and required less time. If MALS is found in a gastrectomy case, median arcuate ligament release may be considered.

## Data Availability

The data supporting the findings of this study are available within the article.

## Abbreviations

MALS: Median arcuate ligament syndrome
MAL: Median arcuate ligament
